# Representing suicide: Giving voice to a desire to die?

**DOI:** 10.1177/13634593211046843

**Published:** 2021-09-20

**Authors:** Ian Marsh, Rachel Winter, Lisa Marzano

**Affiliations:** Canterbury Christ Church University, UK; University of Birmingham, UK; Middlesex University, UK

**Keywords:** death dying and bereavement, ethnography, mental health

## Abstract

Drawing on interview and online ethnographic data from a study of suicide on the railways, this paper describes the ways in which many of the concepts, assumptions and practices of mainstream suicide prevention are challenged in the accounts of those who are planning, or have enacted, a suicide attempt. We reflect on the ethical dilemmas which can arise for researchers (and practitioners) when lived experience accounts diverge – theoretically, morally and in terms of practical implications – from present-day expert ones. In online, ‘pro-choice’ suicide discussions, people describe beliefs, attitudes, ways of thinking and acting which stand in contrast to existing professional and clinical descriptions of suicide and suicidal behaviour. Most obviously, there is often a rejection of ‘pro-life’ positions, which are framed as ideological, oppressive and naïve. For researchers engaging in online ethnography of ‘pro-choice’ spaces, dilemmas can arise in relation to the representation of perspectives which fundamentally challenge not only prevailing norms within suicide research and prevention practice but socio-cultural norms more widely. Similar issues can arise when considering how best to represent research participants when their accounts diverge from accepted ‘expert’ knowledge and beliefs. In-depth qualitative interviews with those who have thought about or attempted to take their own life indicate that existing theories and models of suicide which start from assumptions of deficit and pathology underestimate the extent to which suicide, as the end result of an often-complex series of actions, requires a person to engage in logistical processes of planning, decision-making, imagination and adaptation. The accounts described here, gathered using two different methodological approaches, highlight the ethical issues which can surface when there are competing claims to (expert) knowledge, as well as differences in beliefs, attitudes and moral stance towards life and death. We argue that researchers need to reflect on their own ethical-moral position in relation to suicide, and on the practical consequences of their privileging of some voices at the expense of other, less well represented, ones.

## Introduction

This paper explores some of the theoretical, practical and ethical issues that can arise for researchers when working with first-person accounts of suicide attempts. Drawing on interview and online ethnographic data from a study of suicide on the railways, we describe the ways in which many of the concepts, assumptions and practices of mainstream suicide prevention are challenged in the accounts of those who are planning, or have enacted, a suicide attempt using this method/location, and we reflect on the ethical dilemmas that can surface for researchers (and practitioners) when lived experience accounts diverge – theoretically, morally and in terms of practical implications – from present-day expert ones.

In terms of our own positionality, this article draws on and is situated within the field of critical suicide studies (Button and Marsh, 2019; White, 2017; White et al., 2016). Over the last decade and more, critical suicide studies scholars and activists have endeavoured to reframe approaches to suicide, questioning many of the taken-for-granted assumptions embedded in mainstream prevention practices and attempting to open up the field to a wider range of voices and perspectives (see, e.g. Fitzpatrick and River, 2018; Fitzpatrick et al., 2015; Hjelmeland, 2016; Hjelmeland and Knizek, 2016; Marsh, 2010, 2015, 2016; White, 2017). In particular, there has been a sustained critique of the dominant conception of suicide as primarily an outcome of ‘mental illness’, what one of us (Marsh, 2010) has termed a ‘compulsory ontology of pathology’ of suicide, that has led to the emergence of an ‘individualized, “internalized”, pathologised, depoliticized, and ultimately tragic form of suicide. . . with alternative interpretations of acts of self-accomplished death marginalised or foreclosed’ (p. 43).

Whilst this ‘compulsory ontology of pathology’ has been interrogated and its effects explored, Tack (2019) argues that other aspects of how suicide is habitually framed have been overlooked, with a critique of the notion of ‘prevention’ itself being noticeably lacking from a critical suicide studies perspective. For Tack (2019), a narrative of ‘prevention’ dominates public and professional discourse and practice, and she notes the ‘unquestioned understanding that suicide must be prevented, while the grounds, consequences and effects of such framing remain uninterrogated. In the prevention narrative, life is the natural and normal state against which death is chosen, yet, simultaneously death is constituted as a non-choice in that it is a choice against the natural’ (p. 46). Tack (2019) argues that this is a shared foundational assumption to be found in both mainstream and critical suicidology, and drawing on the arguments of the few voices (see, e.g. Améry, 1999; Baril, 2017, 2018, 2020; Szasz, 1999), that have previously questioned the ‘logic of life’ (Améry, 1999: 13), concludes that ‘[i]n its collective repetition, the desire to live is rendered a natural and originary characteristic of bodies, which means that it is read and lived as a state that all people are by nature individually orientated towards. The choice of death thus comes to constitute a choice against the natural and renders those who choose it unintelligible’ (p. 57).^1^

This paper attempts to address, albeit in a limited way, Tack’s (2019) challenge to properly engage with a critique of prevention as a guiding principle of work around suicide, and to examine some of the ethical and practical aspects that ‘making intelligible’ a desire to die entails. We argue, in line with Tack (2019) and critical suicide studies writers (e.g. Button and Marsh, 2019; Fitzpatrick and River, 2018; Fitzpatrick et al., 2015; Marsh, 2010), that the dominance of notions of prevention and pathology affect researchers’ reading practices of the people they study, and that this in turn impacts on the choices made as to which voices and perspectives are privileged, which are silenced and which are valorised as ‘expert’. This privileging/marginalisation of perspectives can be very stark within suicidology, and we explore the tensions that exist between those people and organisations for whom the goal is always prevention and those individuals or groups with lived experience who reject, implicitly through their actions or explicitly in expressing a desire to die, this stance. We reflect on how researchers represent the concerns, experiences, thought, actions and practices of each group, and where they position themselves in relation to the competing and clearly conflicting values and beliefs that exist between them. Finally, we address some of the issues that emerge around how to give voice to those who want to die whilst being mindful of the social justice issues that make some lives more (un)liveable than others.

Our point of departure for these considerations is a research study which focussed on suicide on the railways. As part of this project, we interviewed 34 people who had tried or thought about rail suicide as a method (Marzano et al., 2019). In addition, we also conducted an online ethnography spanning several online spaces where people gather to discuss suicide, including ‘pro-choice’ suicide forums and discussions. Both forms of data collection and analysis raised ethical issues around representation – which voices did we choose to hear and which we silenced, the interpretation and framing the stories we heard – as well as questions as to where we positioned ourselves as researchers within a contested moral field.

In the following sections we outline the online ethnographic research and then the interviews conducted as part of the study, picking up on salient themes and issues around representation, ‘expertise’ and framing. To begin with, we briefly map the complicated and frequently fraught relationship between ‘pro-choice’ advocates and those who support prevention as it plays out in relation to online suicide discussion forums, as this can help illustrate the contested field researchers and practitioners need to navigate if we are to not simply take up an unreflective ‘pro-life’ stance ourselves. We then look specifically at railway suicide and how this is discussed online by people considering that method, focussing in particular on the challenges to researchers of presenting ‘pro-choice’ perspectives within a field dominated by prevention/‘pro-life’^2^ assumptions and practices.

## Online ethnography of ‘pro-choice’ discussions online

‘Pro-choice’ forums have been around in one form or another since before the World Wide Web, beginning in 1990 with alt.suicide.holiday (a.s.h.), an unmoderated Usenet newsgroup. Starting as a discussion on the possible connections between holiday seasons and suicide rates, the group soon moved on to consider different ‘pro-life’, ‘pro-suicide’ and ‘pro-choice’ views, as well as sharing information about specific methods for suicide, and a community developed of regular participants who identified as ‘ashers’ (Niezen, 2013). With the development of different social media platforms these discussions continued in new spaces as communities continued to seek places where they could converse openly about suicide. However, high profile media coverage of the suicides of a number of community participants included demands for the site to be shut down, a trend which has followed each ‘post-a.s.h.’ iteration of ‘pro-choice’ forums to the present day (Brown, 2016; Love, 2020; Niezen, 2013).

The contrast between the beliefs and aims of prevention advocates and those who identify as ‘pro-choice’ can appear to be stark. In online suicide forums and discussions, there is often a fierce rejection of ‘pro-life’ positions, which are framed as ideological, oppressive and naïve. Perhaps unsurprisingly, prevention advocates have tended to view ‘pro-choice’ forums as being in reality ‘pro-suicide’ and have lobbied for their removal and banning. The positions seem binary, fundamentally opposed and unbridgeable, and the stakes are high for both sides. ‘Pro-choice’ sites contain frequent criticisms of the beliefs of ‘pro-lifers’ – a lack of understanding is taken to be inherent in their attitude to suicide – and of the actions of so-called ‘do-gooders’ which, they argue, impinge on their right (claimed as a universal human right) to end their lives at the time and in the manner of their choosing. Suicide, in ‘pro-choice’ discourse, is coded as an escape, an end to suffering and those who post that they are going to end their lives are, more often than not, wished good luck and a peaceful journey. For their part, those involved in prevention talk of the tragedy of suicide, of such acts not being inevitable, of the possibilities for help that exists and also sometimes allude to the social costs or harm of suicide, such as the suffering involved for those left behind (and many prevention organisations and charities, such as Papyrus in the UK or Roses in the Ocean in Australia, have been set up by people themselves bereaved by suicide or have a significant input from survivors of bereavement by suicide, such as the Zero Suicide Alliance).

However, given that prevention efforts are geared towards (in the broadest sense) helping those experiencing distress and a desire to die to find support so that they can go on living, and that in online forums one frequently finds people who express suicidal thoughts and plans, the two groups potentially relate in ways more complicated than the polarised debate would suggest. Users of ‘pro-choice’ forums often explicitly express a desire to die, and thus could be seen as a ‘target’ population for prevention efforts, but more often than not they are the focus of a more complicated concern, for as well as expressing their own thoughts of suicide, participants also share information with others about specific suicide methods, and sometimes the indirect encouragement of others’ plans through the prevalent idea of ‘respecting a person’s choice’ – be that continuing to live, or ending their own life. It is these aspects of ‘pro-choice’ discussion forums that have frequently drawn media attention and have been used to support attempts to ban such sites (Brown, 2016; Niezen, 2013).

Participants in suicide discussion forums often argue, however, that their community is misunderstood and misrepresented, and that they support others to recover, to seek help, to avoid potentially damaging or traumatic methods, to reduce wider trauma by discouraging actions which involve others (e.g. train drivers). Users also argue that the issues in a person’s life that they feel push them towards suicide are, almost without exception, situated in the ‘real world’ rather than in their online community. In addition, as others have noted, users often comment that the ability to find a supportive community online, even (or perhaps particularly) when the focus is on suicide, can itself have preventative effects (Lundström, 2018; Niezen, 2013; Wiggins et al., 2016).

For researchers tasked with representing such online spaces, and faced with these competing and frequently highly emotionally charged positions, the temptation is to set up what Niezen (2013) characterises as a ‘Manichean dualism’ – that is ‘a struggle of death against life, cultivated despair against rediscovered purpose, pathology versus well-being’ (p. 308) – and to resort to ‘description of the various countervailing pressures that impel those who are at risk of suicide in one direction or another’ (pp. 308, 309). Within such a dichotomised debate it can be hard to find neutral ground, and almost inevitably one side or the other comes to be foregrounded more – either the threat posed by the accessibility of the sites and the dangers of the content is highlighted, or else the preventative effects that arise from the sense of community and belonging often found online are made the main focus.

What is clear, however, is that almost all such discussions inevitably take place against the backdrop of the dominant prevention narrative outlined by Tack (2019), and in relation to what Baril (2018, 2020) characterises as ‘compulsory liveness’ – ‘the injunction to live and to futurity’ – which ‘makes the desire/need for death of some people abnormal, unconceivable and unintelligible, like suicidal subjects themselves’ (Baril, 2020).

The relationship between ‘pro-choice’ and ‘pro-life’ or prevention stances can seem adversarial and oppositional, and as researchers we have often taken the side of ‘life’, ‘futurity’ and prevention without reflecting too deeply on some of the ethical complexities and possible unintended consequences of so doing. Recent research into how railway suicide is constructed in online spaces has given us experience of some of the difficulties and tensions involved in representing online, ‘pro-choice’ spaces, particularly in relation to the compulsory prevention and ‘liveness’ imperatives identified by Tack (2019) and Baril (2020). We will briefly outline this research and describe our efforts to engage with some of the ethical issues involved in the following sections.

## Online ethnography of railway suicide

As part of a wider railway industry funded suicide prevention project, several online spaces were analysed, including forums which centred on suicide discussions and subreddits, which are communities on Reddit created by users around specific topics. These were explored in order to gain insight into why people choose railways as a means and location for suicide. We primarily engaged with the question, ‘Where and why do people take their life on the railways?’ from an online perspective. This allowed us to look in detail at those discourses and background cultural scripts that circulate and influence people’s decisions about whether or not to end their life, and the method and location they consider choosing.

We chose spaces with different levels of moderation, which varied in the degree to which they framed themselves as a ‘pro-choice’ community where suicide methods could be openly discussed, to more moderated spaces where such discussions could be removed. ‘Pro-choice’ discussions on such forums are available on the ‘clearnet’ (as opposed to the ‘darknet’) and provide an abundance of discursive material on specific topics (e.g. different methods of train suicide) not usually available publicly in such detail. They also appear to be widely used by those who want a space to discuss specific suicide methods, and, as such, they can cast light on how suicide on the railways is understood by those who are thinking about, or have tried, to end their life using this method. These communities are composed of people who might not normally get involved in suicide research but whose insights can be very valuable in understanding why people choose the railways for suicide.

In the end we analysed 55 threads on the suicide forum, where the primary focus was on trains or railways as a method/location for suicide. Alongside the original posts, we also analysed the associated conversations which developed through the comments. A typical thread had between 14 and 30 associated comments. We also analysed 199 posts on Reddit where the primary conversation was about railways, and 1228 associated comments. In terms of analysis of the data, a thematic approach was used to identify and analyse patterns of meaning within the texts and fieldnotes. In addition, emerging patterns of interaction in the discussions were noted; that is, the ways in which particular ideas and arguments, points of view, and expressions of emotion recurred within and across threads.

What became apparent through undertaking the research was the complexity of how online discursive spaces relate to any notion of prevention. This can be illustrated by reference to how online discussions usually develop where someone discloses that they are considering suicide on the railway.

Typically, on a ‘pro-choice’ forum or discussion the original poster will usually state that they are considering this method, and sometimes, this will be presented as part of a story outlining what had happened to make them feel suicidal. Reasons for considering the method are given, usually including that it is accessible, affordable, likely to be fatal and likely to be quick. There is often an acknowledgement from the original poster that the method is frowned upon by others on the site as it is considered ‘selfish’ due to the effects on others (e.g. the train driver).

Similarly, people sometimes turn to Reddit to disclose intent and write about their reasons and experiences which have led to them wanting to take their own life by railway. Here, original posters write about difficult situations in their lives, this includes problems at school and work, relationship breakdowns and struggling with life in general. Some posts provide a historical narrative about what has led to this moment of despair. Others are snippets of that day saying, for instance, a recent argument with their parents means that they want to take their own life. Other social media users respond by asking them to talk to them about what they are going through, and help to provide ‘hope’ for the future. People also write of a sense of loneliness or not having others to speak to offline about these difficulties they may be facing and therefore turn to online spaces to share their experiences. They often gain sympathetic responses from a community of people who have, or have had, similar feelings to them.

The disclosure of the desire and/or a plan to end one’s life not involving the railways is usually met on the forum with sympathy for and understanding of the person’s current situation and previous life experiences, a desire for them to find peace with whatever choice they make, and the wishing of good luck (with the exception perhaps of one or two other methods perceived to involve the risk of traumatising others). However, the disclosure of a plan involving the railways is almost always met with a negative response, and the suggestion (often implored) to find/choose another method. On the forum, people generally advise against railway methods for the following reasons:

- It has a traumatic effect on others (especially the driver)
- There is a possibility of surviving with injuries
- There is a risk of intervention
- It is a fear-inducing method so difficult to overcome survival instinct

Occasionally, the fact that the person’s family will have to identify their body is also mentioned.

Interactions on Reddit to disclosure of a plan mirror these responses about the impact it may have on others, and potential of surviving injuries. In response to people writing about wanting to ‘jump in front of a train’, others post reasons not to. ‘Think about the trauma for others involved’ is often used to indicate the impact it may have on passengers, train drivers and the emergency services. People share stories of their friends or family members who have attempted suicide by train before and survived but with long term injuries. Others offer a listening ear and say that they are available to chat to the original poster. ‘I’m here for you if you need someone to talk to’ or ‘talk to me’ are both frequently written within the comments. These arguments and forms of response are often relayed time and time again in threads (albeit in different ways).

Involving others in one’s suicide attempt is often looked upon very negatively in posts, with the potentially traumatic effect on the driver particularly prominent as an argument against the method. Sometimes, the original poster gives reasons why they have to use this method. Often this is because they have tried and ‘failed’ with other ways to end their life or that alternative methods are not available (due to cost, accessibility, etc.). The railway is sometimes presented as a method of ‘last resort’. In many threads, there is considerable (and often heated) debate over whether the method is a ‘good’ or ‘acceptable’ one, and these discussions draw on both practical and ethical considerations.

In terms of practicalities, the arguments for the method being ineffective are often countered by alternative views. As examples, people argue that in order to ensure the method is lethal and to minimise the possibility of surviving with injuries careful planning is required, including consulting research collated on the forums on the specifics of each method of suicide. To reduce the possibility of intervention people are advised to scout the location and to take advice from others on the forum about the best times of day to go, what to wear, how to act and so on. Often, issues around how to overcome the triggering of the survival instinct are discussed, and advice given around managing this through repeated exposure, and the use of alcohol and/or drugs.

The ethics of the method are again openly and frequently discussed, and there are arguments around the extent of trauma to the driver, with some minimising any possible impact on others by rationalising that they will get support, get over it, not be too affected and so on.

Any resolution of these issues in discussion is very rare. There are instances where it has been later reported on the forum that a person has gone on to end their life using the railways, with links to local media reports on the death posted online. People also post that they had considered the railways as a method/location but had changed their mind as a result of the new knowledge or perspective presented to them on the forum.

In terms of understanding how railway suicide is thought about by people considering ending their lives, analysis of the online spaces offered important insights. In particular, the findings from the online research cast light on attitudes to railway suicide amongst an arguably well-informed, ‘motivated’, high-risk group; on who and why people choose the railways as a suicide method/location; the ways in which people try to dissuade others from using railways; the effects of online social pressure to not use the railways for suicide; the effects of increased knowledge on people’s choice of method; how various online platforms are used to discuss suicide in different ways; the ways in which the internet might be changing (as well as reflecting) the prevalence of particular suicide methods; and on the informal peer-to-peer support that people both seek and provide online.

What the research did not provide were easy answers as to whether discussions on social media platforms acted as a preventative force or rather encouraged people to take their own lives – case studies could be constructed of forum users which would illustrate both poles of this continuum, and pretty much all the space in between – or whether any ‘dissuasion’ from rail suicide actually resulted in displacement to other (potentially even more lethal) methods or locations. Niezen (2013) argues that, ‘[s]uicide forums tend to be rigorous, rational, and instrumentally effective when it comes to exchanging information on the techniques of self-inflicted death’ (pp. 304, 305), and whilst for the most part true, that online ‘exchange of information’ is not necessarily straightforward, however. As illustrated by the example of the railways, often the information exchanged is intended to dissuade, or at least to make the person think more carefully about their plans. Other methods are more unambiguously promoted, and the extent to which online discussions relating to ‘techniques of self-inflicted death’ could be said to have either preventative effects or to encourage self-destructive actions does seem to vary across methods.

Once again, however, we are drawn, perhaps inevitably, into considerations framed almost exclusively in relation to prevention.^3^ Whilst the positions of ‘pro-choice’ and prevention advocates could be said to be at points philosophically or ideologically opposed, academic and professional debates on the nature of online suicide discussion forums, and even justifications for the forums themselves by their users, almost always revolve around arguments for the best method for enabling people to go on living – that is, on what works in terms of prevention – and that may include spending time with others discussing suicide and sharing plans to end their life.

What receives little space or attention, certainly in academic texts on suicide, are those voices which unambiguously advocate, often from a first-person perspective, for the right to choose such a death. An important question for researchers, and one we faced when analysing and writing about suicide on the railways, is how to frame and represent those mostly neglected or marginalised voices which veer radically from accepted prevention scripts. Such is the dominance of the discourses of prevention and pathologisation that in academic texts, as well as more generally, suicidal people are almost always framed as ill, impaired or irrational (Marsh, 2010). As Tack (2019) notes, suicide ‘is read from the position of those who are orientated towards life and who, by virtue of citing the desire to live, occupy a position that is viewed as neutral and from which they can read and assess others’ (p. 55), and consequently, those who have a desire to die are, ‘viewed as disorientated, and are rendered pathological and unintelligible, in need of reorientation’ (p. 52). As people who write about suicide, including representing the voices of those who have thought about or attempted to take their own lives, we often, consciously or not, take up such a life-oriented position, and from here ‘read and assess others’, often through the lenses of pathology and prevention.

This framing and researcher-positioning can be difficult to resist, and it also emerged as a concern in relation to interviews with attempt survivors conducted as part of the study. In the next section we briefly outline the research which focussed on interviews with survivors of attempts on the railways, and reflect further on the ways in which the framing of suicide as pathological and unintelligible can influence the reading practices of researchers. Common themes from both elements of the research are then explored in more detail, particularly in relation to the ethical issues raised in foregrounding the voices of those who express a desire to die and who advocate for the right to do so unimpeded. An argument is made, from a critical suicide studies perspective, that we also need to take account of the social justice issues that make some lives more (un)liveable than others in our framings of both ‘pro-choice’ and prevention positions.

## Giving voice to suicide in interviews

The issues that can arise when attempting to give voice to suicidal people in a non-pathologising way can be illustrated by reference to interviews we conducted and analysed as part of the railway study.

We interviewed 34 UK-based men and women aged 18 or over who had either survived a suicide attempt on the railways, survived a suicide attempt by another method (having considered but rejected a rail suicide), or experienced thoughts of rail suicide but not made a suicide attempt. The qualitative interviews explored in depth the lived experiences of considering and/or attempting suicide specifically by train. The primary aim of the interviews was to give participants free reign to describe in their own words the processes they went through in planning and undertaking a suicide attempt.^4^

When analysing the interviews, we were aware that such accounts are usually framed in relation to concepts of deficit and pathology. Existing models of suicidal behaviour frequently focus on the psychology of the ‘suicidal mind’,^5^ and often on impaired individual cognitive processes. Specifically, suicidal thoughts and plans (such as choice of method or location) tend to be read as arising from limited or constricted individual thought processes and reasoning. Deisenhammer et al. (2016), for instance, talk of ‘increased cognitive rigidity’ (p. 15) and a ‘reduced potential to plan the suicidal act in a way that maintains the possibility to adapt one’s actions to changing circumstances’ (p. 213). O’Connor and Nock (2014), summarising psychological research on suicide, describe the ‘different cognitive processes that might be deficient or dysfunctional in suicidal people: ‘For decades, clinical and theoretical accounts have described suicidal people as being cognitively rigid or inflexible’; ‘[i]mpaired decision making is also evident in suicide attempters’; ‘a tendency to suppress unwanted thoughts’; ‘a decreased ability to recall specific autobiographical memories, which might in turn impair their ability to imagine the future and to engage in effective problem solving’; ‘study findings have consistently shown a link between suicidal behaviour and deficits in both interpersonal problem solving and coping’; and ‘impaired positive future thinking’ (pp. 77, 79). For Joiner et al. (2016), the ‘calculation of the value of one’s own death exceeding the value of one’s life. . . represents a tragic, flawed and sometimes fatal miscalculation (i.e. a derangement)’ (p. 243).

In addition, given the tendency in academic and professional research and writings to see mostly pathology and deficit in the functioning of people planning to end their life, it is perhaps unsurprising that suicide attempts are often characterised as ‘impulsive’, involving only limited preparation. For example, Williams and Wells (1991) suggested that two-thirds of suicide attempts were contemplated for less than an hour beforehand, and Florentine and Crane (2010) concluded that ‘a substantial proportion of suicide attempts are not planned far in advance, even when the method of choice is highly lethal’ (p. 1628).

When we came to read through the interview transcripts, however, evidence of impairment, rigidity and impulsiveness were not so apparent. Instead, individuals described engaging in a complex activity or set of tasks involving planning, choice, visualisation and adaptation. People recounted that they imagined and rehearsed in their minds particular scenarios involving their own death and the impact it would have on others, often constructing a detailed story about their suicide attempt. Participants also described a dynamic, iterative relationship between themselves and their cultural, social and physical environments before, during and after an attempt.

We could, undoubtedly, have framed the accounts by drawing on notions of deficit and pathology – for example, ‘increased cognitive rigidity’ and ‘impaired decision making’ could be inferred at times in the sense that planning to end one’s life might indeed require a determination that could be read as rigidity, and the fact of the desired or expected outcome being death could of course (as it usually is) be described as an outcome of impaired decision making. Similarly, going over possible actions in one’s mind (‘rehearsing’ or imagining events) could be considered a form of rumination or cognitive compulsion.

There were also, inevitably, processes of co-construction and the influence of socially-available dominant cultural scripts in participants’ accounts. Interviewees would have been asked to recount their story to numerous other people (e.g. emergency workers, doctors, nurses, therapists, families, friends and so on), and through this process a version of events may have evolved that leaves out uncertainties or difficult admissions (such as planning to act in ways that had the potential to cause harm), and which involve ‘after the event’ reconstructions or rationalisations. Through these processes discourses of prevention and pathology could have been drawn on in the formation of these accounts, and the interview approach we used may also have played a part in the creation of a particular narrative. We did, however, strive to ensure as much as possible that participants were given the time and space to recount their stories in their own words and in their own way.

Even taking into account these processes of co-construction, suicide emerged in participants’ accounts less as an impulsive act of a person experiencing acute mental illness or cognitive impairment, and more as the end result of a series of actions requiring planning, decision-making and choices, adaptability and imagination (and often a series of disappointing, at best, interactions with deficient and dysfunctional services, within a context of demoralising and distressing life circumstances and events). It was often described as a process whose beginnings stretched back a considerable length of time. So, whereas existing academic and professional accounts tend to focus on particular cognitive aspects and states of the suicidal person, in the first-person accounts gleaned from the interviews a suicide attempt is framed much more as a physical and logistical problem, almost as a project requiring careful planning and forethought to be ‘successfully’ completed.

In the end we resisted falling back on familiar tropes of pathology and deficit in our analysis and write-up of the interviews, relying instead on the descriptions and narratives provided by participants and limiting any additional layers of interpretation. The rationale for this was mostly practical, in that we felt there is a danger in understanding suicidal thoughts and behaviours as arising primarily or solely from psychological deficits or dysfunction, and that an unnecessarily narrow (and potentially distorted) view of the processes and planning involved in a suicide attempt is produced through such means. Again, though, a prevention imperative was possibly at work here too, in that our thinking was shaped towards understanding the participants’ experiences in order to inform preventative interventions.

## Liberation?

Constructing accounts of suicide and suicidality that run counter to the prevailing prevention narrative and accepted socio-cultural norms around such experiences, desires and forms of death is challenging. As Tack (2019) notes, prevention is constructed as the ‘pre-discursive truth of suicide outside the realm of what can be questioned’ (p. 50), and thus it can be difficult to find an acceptable language with which to articulate ‘pro-choice’/‘pro-suicide’ perspectives. Indeed, such is the dominance of the prevention imperative that it can even be hard to grasp what possibilities for thought and action exist outside of its reach. These difficulties are faced by researchers but also surely more acutely by those experiencing such a desire to die, who are routinely taken to be irrational and ‘other’. For Baril (2020), the silencing and marginalisation of the views and experiences of suicidal people, and the lack of access to the theoretical tools needed to understand and explain the oppression they experience, represents forms of violence and injustice, which he names as ‘suicidism’. Drawing on Fricker’s (2007) notions of testimonial and hermeneutical injustice, Baril (2020) argues that the ‘judgment of suicidal people as irrational, incompetent, illegitimate or alienated’ destroys the credibility of suicidal subjects and invalidates their voices.

Whilst giving voice to the suicidal is surely a defensible aim from an anti-oppressive perspective, the charge can still be levelled that in so doing one is normalising, or even validating, suicide itself. When faced with the reality of a death by suicide (which we have in different ways), there is then often a resistance to those ‘pro-choice’ arguments that rest solely on an appeal to individual rights and ideas of ‘non-interference’. The trauma and harm to others (noted frequently, to be fair, both online and by the study interviewees) is impossible to dismiss entirely when considering the ethics of suicide.

Alongside such considerations, it is also important to acknowledge that prevention approaches that rest on notions of irrationality and pathology as explanations for suicide often seem to fail to meet the needs of many people in crisis and distress, or those who live with chronic suicidality (Delano, 2013; Webb, 2010). For Baril (2020), a solution of sorts, and a kind of bridge between prevention and acceptance or validation of a desire to die, is ‘suicide-affirmative’ healthcare. Here, a non-coercive approach is advocated, one that,

‘would offer care and support through an informed-consent model, taking for granted that the expert in the decision to transition, in this case from life to death, is the person making the decision. It goes without saying that before implementing this approach, it would be important to engage in extensive critical reflection regarding the conditions, regulations, safeguards, and type of accompaniment, as well as the simultaneous sociopolitical changes necessary, to reduce suicidal ideations. These concrete aspects of a harm-reduction approach would have to be determined primarily *by and for* suicidal people and their allies, mobilizing their expertise on suicidality’.

There is much to admire in Baril’s (2020) formulation, such as the foregrounding of the wishes, needs and expertise of suicidal people in any decision-making process and the acknowledgement of the necessity of first creating ‘safer spaces’ within which deliberations and ‘critical reflection’ can occur, ones that, ‘must be as free as possible from forms of judgment, stigmatization, paternalism and oppression and must foster a welcoming environment so that suicidal people can freely express their lived experiences, thoughts and demands without fear of reprisals and negative consequences’.

Interestingly, the online spaces we encountered in our research often seemed to fulfil these criteria, but perhaps the freedoms offered by such places also need to be weighed against an acknowledgement that deaths by suicide can follow. For Baril (2020), alongside the opening up of new thought and practices around suicide would have to be an acceptance that, for a small number, suicides would occur. Such deaths, however, Baril (2020) argues, would be less traumatic for all involved, with people determined to die able ‘to carefully plan their death several weeks or months in advance, to say goodbye to their loved ones and to leave this world using less lonely and violent means than those usually employed in completed suicides’.

The possibility remains, however, that any such approach would not fully escape the forms of power that constitutes and produces suicides according to a pernicious social logic (Button, 2016; Marsh, 2019). The interactions between social structures, hierarchies, and moral economies of human worth, the psychic and emotional life of people caught up in such regimes, and deaths by suicide are unlikely to cease to function (Marsh, 2019; Mills, 2018), and the formation of ‘suicidal subjectivities’ over time within unjust systems would continue but be masked by approved processes of consent. The concern would be that any such model would not, in reality, represent a genuine form of liberation.^6^

## Building bridges

As researchers, we encounter a heterogeneity of voices, perspectives and attitudes in relation to suicide. They are often, for obvious and understandable reasons, emotionally charged and thus rarely neutral. Questions as to which voices and perspectives we privilege, which we silence, which we valorise as ‘expert’,^7^ and our attitude towards those who don’t want to be heard by ‘us’, are central to any exploration, as is a consideration of the effects, intended or otherwise, of foregrounding certain stories over others. Critical suicide studies scholars have explored the relationship between dominant narratives, relations of power and the effects of framing stories of suicide in particular ways (e.g. Fitzpatrick, 2016; Fitzpatrick et al., 2015; Fitzpatrick and River, 2018; Marsh, 2010, 2019; White and Morris, 2010), and this work has highlighted the ways in which discourse, politics and experiences are intertwined. As Fitzpatrick (2016) argues, ‘personal stories of suicide confer certain privileges and benefits on survivors of suicide attempts, they also manifest and normalise particular ways of thinking, acting and communicating that have considerable ethical and political force in shaping the ways suicidal behaviour is understood, the ways it is subjectively experienced and the ways it is responded to’ (p. 267). Analysis of such complex relations opens up possibilities both for understanding more clearly the effects of the prevention imperative and also for us to begin to think beyond the dichotomised framings of either ‘pro-life’ or ‘pro-choice’ positions.

Such a task isn’t easy, however, and for researchers there is perhaps no easy space to occupy. As Niezen (2013) articulates, the temptation is to accept and take up a position within a polarised debate that sets life versus death, hope versus despair, rationality and sanity versus irrationality and pathology. In online spaces, the prevention/‘pro-choice’ debate certainly appears oppositional and adversarial; on one side, suicide is coded as death, trauma, tragedy and grief and on the other it is read as escape peace and relief. Similarly, ‘prevention’ for one group is associated with notions of protection, intervention and the primacy of life, and for the other (particularly those expressing in online forums a desire to die) it is interference, an infringement of rights and an unwarranted ‘external’ demand for the continuation of suffering.

Whether the apparent gaps or distance between ‘pro-life’ and ‘pro-choice’ positions can – or indeed should – be reconciled is of course unlikely to lend itself to a simple or singular solution. Bridging, rather than ‘resolving’, these positions may however help navigate the complex ethical and practical challenges discussed in this paper – in better informed, if not necessarily more ‘effective’, ways.

A necessary step in any bridge building would be an acknowledgement (from prevention advocates) that suicide prevention itself is a morally contested field, and this would rest on accepting as legitimate voices currently marginalised or silenced as irrational and pathological. There would be practical, as well as ethical, reasons for doing so; one can ask how effective any interventions designed to prevent suicide can be when the beliefs, attitudes, ways of thinking and acting of the ‘target’ population are so far removed from the assumptions and practices of those engaged in prevention. What was very noticeable in our research was the extent to which interviewees and those participating in online forums were often dismissive of initiatives taken to be ‘pro-life’. As an example, a common prevention approach is to encourage help-seeking behaviours; yet participants, particularly online, often positioned themselves as what could be called ‘post help-seeking’ in that they recounted numerous attempts to get help (from mental health services, charity helplines, etc.) but had not found them useful and would not be using them again.

The disconnect between the assumptions and beliefs of prevention advocates, and those people expressing a desire to die, often appeared to be quite stark in our research, and the deployment of notions of irrationality, pathology and impairment to frame ‘pro-choice’ positions can further exacerbate this divide. At the same time, however, formulations of suicide outside of prevention discourse can themselves be problematic, often resting on ideas of individual free will, choice and rights that can sit uneasily with our understanding of the social and political determinants of suicide (Button, 2016, 2019) and the known social impacts of such deaths (Pitman et al., 2014).

It is, however, important to recognise and reflect on the diversity of voices in terms of their ‘continuous’ nature – above and beyond pro-choice versus pro-life stances (and the continuum on which they may in turn rest). One learning from the railway project was the understanding that the seemingly polarised debate occurs at a certain level of discourse, and that more subtle variations, tensions and contradictions are discernible when looked for. For example, people bereaved by suicide and those supporting traditional preventative approaches can themselves live in relation to their own thoughts of suicide, and people with a desire to die can also, at the same time, have a desire to live. Indeed, whilst it is important to bring attention to the potential divide – and power imbalances – between prevention and ‘pro-choice’ views, this very dichotomy risks reinforcing monolithic (mis)constructions of both as mutually exclusive categories, which they are clearly not.

The accounts described here, gathered using two different methodological approaches, highlight the ethical issues which can arise when trying to navigate and represent a contested moral field, where fundamental differences in beliefs, attitudes and stances towards life and death co-exist. For researchers, there may be conflicts and dilemmas in relation to representing perspectives which fundamentally challenge not only prevailing norms within suicide research and prevention practice but socio-cultural norms more widely. We argue that as researchers we need to reflect on their own ethical-moral position in relation to suicide, on which voices we choose to privilege and how we represent those voices that reject or question mainstream prevention assumptions.

## Notes

1 See also the work of Baril (2017, 2018, 2020), who argues that the ‘injunction to live’ (Baril, 2017: 201) is at the heart of all prevention efforts.

2 ‘Pro-life’ or ‘pro-lifers’ are terms often given to prevention advocates (and to people who oppose suicide more generally) on ‘pro-choice’ discussion forums.

3 The project itself was funded and framed as aiding prevention efforts on the railways.

4 See Marsh et al. (2021) for more details.

5 For example, the Integrated Motivational-Volitional Model of Suicidal Behaviour (O’Connor, 2011; O’Connor and Kirtley, 2018).

6 Though one can argue in a similar way to Foucault (1981) that the act of suicide itself represents an escape from all forms of power.

7 The notion of ‘expertise’ in suicide prevention has been eloquently critiqued by, amongst others, Webb (2010) and Delano (2013) from a lived experience perspective.
